# Speech and music shape the listening brain: evidence for shared domain-general mechanisms

**DOI:** 10.3389/fpsyg.2013.00321

**Published:** 2013-06-04

**Authors:** Salomi S. Asaridou, James M. McQueen

**Affiliations:** ^1^Donders Institute for Brain, Cognition and Behaviour, Radboud University NijmegenNijmegen, Netherlands; ^2^Max Planck Institute for PsycholinguisticsNijmegen, Netherlands; ^3^Behavioural Science Institute, Radboud University NijmegenNijmegen, Netherlands

**Keywords:** speech, language, music, auditory processing, domain-general processes, interaction, transfer effects, brain and behavior

## Abstract

Are there bi-directional influences between speech perception and music perception? An answer to this question is essential for understanding the extent to which the speech and music that we hear are processed by domain-general auditory processes and/or by distinct neural auditory mechanisms. This review summarizes a large body of behavioral and neuroscientific findings which suggest that the musical experience of trained musicians does modulate speech processing, and a sparser set of data, largely on pitch processing, which suggest in addition that linguistic experience, in particular learning a tone language, modulates music processing. Although research has focused mostly on music on speech effects, we argue that both directions of influence need to be studied, and conclude that the picture which thus emerges is one of mutual interaction across domains. In particular, it is not simply that experience with spoken language has some effects on music perception, and vice versa, but that because of shared domain-general subcortical and cortical networks, experiences in both domains influence behavior in both domains.

## Introduction

There are two ways to approach the comparison of language and music: either by providing a long list of their differences, or a surprisingly long list of their commonalities. In recent years, the latter way has been far more popular than the former. This is not an attempt to underrate the uniqueness of each domain in the human cognitive repertoire. Language and music are undoubtedly systems with distinct representations, structure, and utility. Nevertheless, commonalities do emerge when one considers that they share the same basic building blocks. For both perception of speech and perception of music, the starting point is the temporally organized acoustic signal (Besson et al., 1997; McMullen and Saffran, 2004; Patel, 2008). Despite the fact that speech primarily makes use of timbral while music makes use of pitch contrasts, pitch information is also relevant to speech, and timbral contrasts are also used in music, whilst both organize the acoustic signal in distinct sound categories (Patel, 2008).

One might nonetheless wonder: why is a comparative approach to language and music interesting? After all, no matter what the similarities are, a jazz improvisation piece will always be easily distinguishable from a homily. Apart from purely theoretical reasons for taking a comparative approach (see Besson and Schön, 2011), a great incentive for emphasizing the shared properties of language and music stems from accumulating evidence showing that experience with one of the two induces plastic changes to the brain's structure and function. It has been long argued, for example, that the musician's brain provides a model for plasticity (Münte et al., 2002). What has drawn even more attention to this topic is the fact that extensive music training enhances auditory processing not only within but also beyond this domain, to general auditory and speech processing. This finding is of great value to our understanding of auditory perception mechanisms and their plastic properties. In particular, it indicates that at least some auditory mechanisms are domain-general in nature, and thus are not special to either music or speech processing.

The spotlight of attention so far has been mostly on the effects of musical training and experience on linguistic processing. However, there are two terms in the music and language equation, and although focusing on the consequences of music experience on speech is justifiable, it is of equal importance to investigate what happens when the terms are reversed. Indeed, given the aforementioned similarities between the two domains and the idea that influences of music on speech arise because of shared, domain-general auditory mechanisms, it is likely that linguistic experience will have an effect on music processing. Asking whether the influences are bidirectional thus offers an important test of the claim about domain-general processes and should help to define where in the processing stream those general mechanisms end and where domain-specific mechanisms begin.

In the present paper we will review the evidence for bi-directional influences between speech and music. While language and music influence each other at multiple levels from sounds and melodies to semantics and syntax, in this review we will focus on the level of sound processing. We begin by summarizing the extensive evidence on the effects of musical experience on linguistic sound processing and then discuss existing theoretical frameworks that seek to explain these data. That discussion leads to the theories' predictions concerning the effects of linguistic experience on musical behavior, and then a review of the smaller body of findings about such effects. We will discuss behavioral data and describe the brain structures which appear to be involved in music and speech processing, making the case that there are resources shared across domains. We also cover the evidence on mutual interactions between speech and music, as well as on structure-function associations in the brain. We then discuss the challenges that will need to be faced by future research in this area. We conclude that there is convincing evidence that speech and music interact in shaping the auditory brain and in jointly determining aspects of perceptual behavior in both domains.

## Effect of music on speech

There is a wide range of research focusing on the effect of music on speech processing. At the behavioral level, there is evidence that musical aptitude correlates uniquely with L2 phonological production and perception abilities in adults (Slevc and Miyake, 2006) as well as in children (Milovanov et al., 2008). This correlation between the ability to perceive, discriminate and process music sounds, on the one hand, and the ability to perceive and pronounce non-native speech sounds in musically naïve individuals on the other, suggests that common processing mechanisms mediate both. Musical aptitude can also predict performance in linguistic tone discrimination task in non-tone-language speakers (Delogu et al., 2010). Non-tone-language speakers that score highly on melodic perception tasks also score higher in tonal discrimination tasks (Delogu et al., 2010). Furthermore, individuals with music training are better in discriminating and imitating Mandarin tones than non-musicians, even when the task requires categorical rather than pure auditory perception (Gottfried et al., 2004). This performance superiority cannot be attributed to absolute pitch abilities (Lee and Hung, 2008).

Domain-general sound processing abilities have been found to be predictors of lexical tone learning performance (Wong and Perrachione, 2007; Wong et al., 2008). Participants' performance in a non-lexical, pitch contour identification task was predictive of their ability to use pitch in a Mandarin-like word learning paradigm (Wong and Perrachione, 2007). Thus, the ability to perceive and represent pitch movement which is important in music facilitated learning lexical tone, or linguistic pitch patterns. These findings support the view that speech processing depends, at least in part, on domain-general processes shared with music.

Subcortical auditory processing is also shaped by music training. Information about the malleability of subcortical auditory processing mechanisms primarily comes from electrophysiological studies using the FFR (Frequency Following Response) component. FFR is a brain-wave that is elicited preattentively and originates in the inferior colliculus in the rostral brainstem. It encodes the waveform of the *f*0 of an auditory stimulus in a phase-locked manner (Worden and Marsh, 1968). Wong et al. (2007b) compared FFR responses elicited by musicians and non-musicians while listening to linguistic pitch patterns. They found that musicians' FFR responses followed pitch contours with greater fidelity than non-musicians'. In addition, musicians' auditory brainstem responses encode spectral characteristics of the speech signal (vowel formants) with greater precision compared to non-musicians when participants are listening to degraded speech (Parbery-Clark et al., 2009; Bidelman and Krishnan, 2010). Such enhancement of subcortical encoding of formant characteristics in speech can take place as early as 3 years of age, according to a recent study (Strait et al., 2013). Long-term domain-specific training can therefore augment subcortical sound processing mechanisms (Wong et al., 2007b). This superior subcortical neural representation of speech and music stimuli correlates positively with the amount of music training received, suggesting that it is primarily shaped by experience rather than innate abilities (Musacchia et al., 2007).

Extensive training and experience with music leads not only to subcortical changes but also to plastic changes in the activation of the cerebral cortex, possibly by sharpening cortical preattentive and attentive pitch processing networks. When presented with speech, musicians showed higher bilateral middle temporal gyrus activation compared to non-musicians (Oechslin et al., 2010). The higher the training in music, the lower the activation found in primary auditory cortex (PAC) areas, an indication of more efficient processing of acoustic information (Oechslin et al., 2010). Schön et al. (2004) used an EEG paradigm in which the *f*0 of the final syllable of a sentence was manipulated to create prosodically incongruous stimuli. Musicians showed advanced pitch contour processing of the sentences, as reflected by task performance accuracy as well as EEG recordings (Schön et al., 2004). Similar results are obtained when comparing children who have received musical training to their musically naïve peers (Magne et al., 2006). Facilitated cortical pitch processing, as revealed by EEG data, in both music and language found in these “early musicians” constitutes positive transfer from one domain to the other. A longitudinal study by Moreno et al. (2009) controlled for existing predispositions in “early musicians,” or effects of cognitive motivation and/or maturation that might have affected the results mentioned above. Children randomly assigned to receive music training outperformed their matched peers who had received an equally intense and interesting painting training, both in terms of accuracy but also in their electrophysiological responses to speech stimuli (Moreno et al., 2009). Moreover, this enhancement is not limited to native language processing but extends to foreign languages as well. French musicians were faster and more accurate than non-musicians in detecting prosodic pitch violations in Portuguese, a language not spoken by either group (Marques et al., 2007).

It has been shown that musical training not only facilitates lexical tone processing but also segmental processing, such as for example the processing of consonants (Marie et al., 2011). Interestingly, these facilitation effects cannot be merely due to attention (Marie et al., 2011). Musicians outperform non-musicians in phonetic categorization and their superior performance is associated with higher left Planum Temporale (PT) activation (Elmer et al., 2012). In addition, musicians' electrophysiological responses to phonetic cues such as Voice Onset Time (VOT) (the time between the release of articulatory closure and initiation of voicing) differ from non-musicians', although no differences are detected in behavioral performance (Ott et al., 2011). This advantage was further observed in children who, after being randomly assigned to a musical training group, improved in VOT and syllable duration processing with 1 year of training (Chobert et al., 2012). After 2 years of music training, children also improved their speech segmentation skills (François et al., 2012). Because the children were randomly assigned to the music training group and because of the longitudinal design, it can be concluded that the beneficial effects are due to the training and not pre-existing differences between groups.

This plethora of evidence showing that music training or aptitude can influence linguistic behavior casts doubt on whether music and speech are fully modular, encapsulated systems. Data from double dissociations in neuropsychological patients (i.e., patients with preserved speech production or comprehension but impaired tonal pitch abilities, and patients with spared tonal or singing but impaired speech abilities) previously led to the conclusion that music is subserved by components that are domain-specific and neuroanatomically distinct (Peretz, 2006, 2009). Peretz and Coltheart (2003) have proposed such a model in which a domain-general “acoustic analysis” module is the first to receive and process the acoustic input. Depending on the nature of the input, this module feeds it forward to a music-specific module (“contour analysis”), to a language-specific module (“acoustic-to-phonological conversion”), or to a module which has not yet been confirmed to be either musical or linguistic (“rhythm and meter”) (Peretz and Coltheart, 2003). Although this model assumes that there is a common acoustic processing module, its role is not well defined and only forward flow of information from that module to further processing nodes is allowed. The literature reviewed above, however, suggests that there are either feedback connections from music processing levels to basic acoustic processing levels or direct connections between the domain-specific modules. With compelling evidence against strict modularity increasing, a number of theoretical frameworks that can account for language-music relationships have emerged.

## Theoretical frameworks

We have reviewed behavioral, cortical and subcortical data showing that music training influences linguistic processing. How can these effects be explained? Several frameworks have been proposed, either referring to shared mechanisms between music and language, or even going beyond that to explain how transfer phenomena occur.

### Sharpening of shared auditory skills

One of the most parsimonious accounts for transfer effects is one where music and language share the same auditory processing infrastructure. The argument made is that as this infrastructure becomes more efficient as a result of music experience, this leads to more efficient speech processing. The basic assumption is that the auditory system is malleable and changes with experience. This is supported by a variety of evidence ranging from animal studies to sensory deprivation and perceptual learning effects in humans (for a review see Kraus and Banai, 2007). The fact that music training retunes sound encoding even at its most basic subcortical level reinforces the view that domain-specific experience sharpens domain-general auditory mechanisms (Kraus and Banai, 2007; Kraus and Chandrasekaran, 2010; Skoe and Kraus, 2012). It is proposed that music training enhances these skills primarily through top-down feedback connections from cortical to subcortical sound encoding structures (Kraus and Chandrasekaran, 2010). Musicians learn to guide their attention to meaningful information in the acoustic signal, which in turn leads to improved sensory encoding of this information. Considering the overlap between the acoustic and cognitive demands for music and language, it has been suggested that similar listening skills are required for processing both of them, and hence to the observed transfer effects (Kraus and Chandrasekaran, 2010).

### The shared sound category learning mechanism hypothesis (SSCLMH)

According to Patel (2008), music and language make use of domain-specific categories which exploit different attributes of sound. However, it is hypothesized that the mechanism for sound category learning is common across the two domains. The influence of music training on language can therefore be attributed to the sharpening of an underlying domain-general sound learning mechanism. Patel proposes that statistical learning could be such a mechanism, serving both domains and being indifferent to the nature of the final product that is, to the characteristics of the acoustic signal being exploited. Such a domain-general learning mechanism for language and music has also been put forward by McMullen and Saffran (2004). While reviewing data on the ontogeny of language and music in human infants, they conclude that both domains rely on the same learning mechanisms, namely extraction of an abstract set of rules through statistical learning, in order to form “native” sound categories (McMullen and Saffran, 2004).

### Beyond shared mechanisms

Besson et al. (2011a,b) agree that there is a common mechanism processing the same acoustic parameters in speech and music. If long-term experience with music only sharpened shared acoustic processing abilities in language, then this would indicate that a domain-general processing mechanism account would suffice. However, in order for a theoretical account to be complete, transfer effects should be taken into consideration. If long-term experience in one domain not only sharpens common characteristics but also domain-specific characteristics, this would indicate that experience can transfer from one domain to the other. Evidence in favor of this account should demonstrate that experience in music should facilitate not only domain-general but also domain-specific processing in language. The fact that musicians are better in segmental processing of a non-native language (Marie et al., 2011) is an example of transfer as defined in this framework.

Lastly, Patel's OPERA hypothesis builds up on Kraus and Chandrasekaran's (2010) account, in order to specifically explain how music training facilitates subcortical speech processing (Patel, 2011). Although this hypothesis is mainly concerned with the effect of music on brainstem plasticity, it can serve as a framework for other levels of plasticity pertaining to music and speech. “OPERA” is an acronym composed from the initial letters of five conditions necessary for transfer to occur. These, according to Patel, are the following: (1) Overlap, the fact that training has to tap into a common neural circuit for music and speech, (2) Precision, the demands for processing precision should be high in order to trigger top-down tuning, (3) Emotion, refers to the importance of the emotional rewards that music offers, (4) Repetition, the simple learning principle which is a *sine qua non* for plasticity to occur, and (5) Attention, refers to the importance of engaging focused attention while training. According to the OPERA hypothesis, whenever those prerequisites are fulfilled, music training induces plastic changes that can in turn impact speech processing (Patel, 2011).

## Can language experience have an effect on music? predictions deriving from the theoretical frameworks

None of the above frameworks assumes that the influence of music on language should be unidirectional. On the contrary, bidirectional influences are inherent in shared auditory skills accounts, since they attribute the effects of music on speech to the sharpening of skills mediating both domains. If this mechanism (a common auditory processing or learning mechanism) is shared between music and language, language experience should influence music perception. However, each account makes different predictions with respect to how these influences can occur.

According to the shared auditory skills accounts, language experience can and does induce plastic changes to auditory processing and through that to music processing (Kraus and Banai, 2007; Krishnan et al., 2012). Nonetheless, it could be argued that these changes would mostly result from bottom-up statistical learning instead of the top-down nature of learning in music, and might also be more dependent on sensitive periods. The same holds for the SSCLMH (Patel, 2008). Patel (2008) states that there is, as yet, no evidence against the possibility that the mechanism for sound category learning is common across the two domains. Any experience or training that would increase the efficiency of the sound category learning mechanism should be beneficial for both music and language. It should be noted that contrary to the shared auditory skills accounts, the SSCLMH predicts that individuals with either music or linguistic experience should be better in learning new sound categories. It is therefore not automatically assumed that a domain-general sound processing device improves and manifests itself in music and language but rather that the learning device is more resourceful, and this can only be manifested when new learning is required.

Things get more complicated with frameworks that go beyond shared resources and attempt to include transfer effects in their interpretation of music-language interactions. Although bidirectional influences are not ruled out, and although in theory transfer effects from language to music should be possible, the thresholds for these effects to be detected become higher. That is, the demands on language experience or training are higher. Let us consider the OPERA hypothesis, for example. As summarized above, there are five conditions that have to be met in order for language to affect the neural encoding of music, at least in a subcortical processing level (Patel, 2011). The Overlap and Repetition conditions are assumed to be met in an individual who speaks a tone language. However, the Precision, Emotion, and Attention conditions might not be met, at least not in the same way as they would be met in music training. Although precision is required for using pitch in a tone language, the demands are not comparable to those for music. There is experimental evidence that pitch is neither necessary nor sufficient for speech perception: Mandarin is intelligible even in the absence of pitch variation (Patel et al., 2010) while plenty of contextual and grammatical cues are available in the signal aiding speech comprehension (Xu, 1994; Liu et al., 2012). This difference in precision demand is very important for plasticity-induced fine tuning of the auditory system to take place (Patel, 2012). If the precision demands on auditory encoding placed by music are much higher than those placed by speech perception, one should expect no or very weak effects of language experience on music processing (Patel, 2012). With respect to the rest of the OPERA conditions, it is difficult to define how emotionally rewarding speaking a tone language can be. Although language is a vehicle for communication of emotions, that alone does not automatically mean that the emotion criterion is satisfied. Lastly, the demand for focused attention is one that cannot be met when language experience is defined as tone language experience. Although focused attention is imperative for music training, if not with respect to sounds, then certainly with respect to motor coordination, language acquisition is something that happens effortlessly and naturally (Kuhl, 2004). Under these assumptions, one would have to define language experience differently, in order to observe transfer phenomena. Some alternatives would be to look at trained phoneticians, multilingual individuals, or simultaneous interpreters (see Elmer et al., 2011) where precision, focused attention and executive control are important in a manner more comparable to music.

Despite the fact that defining language experience and finding its effects might be more complicated in comparison to music, there are no theoretical reasons to exclude this possibility. In the following section we provide an overview of studies that have examined effects of language experience on music and sound processing. Evidence is presented according to different processing levels, ranging from behavioral to brain structure studies.

## Evidence of bidirectional influences

### Behavioral evidence

There is clear behavioral evidence of bidirectional influences between speech and music. In an earlier section, we discussed musicians' superior processing of segmental and subsegmental VOT speech cues. What was not mentioned, however, is the fact that perception of acoustic features is not enhanced equally but instead interacts with linguistic experience. In a cross-linguistic experiment with Japanese and Dutch speakers, Sadakata and Sekiyama (2011) showed that although discrimination and identification of non-native temporal and spectral speech contrasts (Japanese consonants and Dutch vowels respectively) was better in musicians, there were stimuli for which musicianship had no advantageous effect. This, according to the authors, is a constraint posed by linguistic experience, namely the effect of a change in the weighting of perceptual cues as individuals develop their native language categories (Sadakata and Sekiyama, 2011). Linguistic influences are thus already present in the studies on musicians. Linguistic experience interacts with music experience, shaping and restricting the perception of the acoustic signal.

Whether domain-specific experience with language has domain-general consequences has been partially addressed by studying tone language speakers' ability to process pitch in a non-linguistic, musical context. It appears that tone language speakers' fine-grained pitch processing ability can transfer to music. When tested in music perception, speakers of Mandarin outperform English speakers in detecting contour and interval changes in simple melodies (Bradley, 2012) while speakers of Cantonese are better than English speaking non-musicians in melody discrimination and tonal memory (Bidelman et al., 2013). Tone-language speakers perform better than non-tone-language speakers in musical interval production and perception tasks (Pfordresher and Brown, 2009) as well as in pitch discrimination tasks (Guiliano et al., 2011; Bidelman et al., 2013). This superiority is more pronounced in small pitch excursions (Guiliano et al., 2011) but not when these are much smaller than the excursions occurring naturally in the respective tone language (Bidelman et al., 2013).

Experience with a tone language seems to provide a perceptual attunement to pitch contours (Stevens et al., 2011). Thai speakers outperformed native English speakers in discriminating contours in speech and filtered speech, in both Thai and English. They were also faster than their control group in detecting contour characteristics in music stimuli (Stevens et al., 2011). Another study, however, found that tone-language speakers were significantly worse than non tone-language speakers in detecting downward pitch differences in simple melodies (Peretz et al., 2011). Since this disadvantage occurred only when the direction of the interval was descending, the authors claim that it is signaling interference from language experience (falling tones in Mandarin are larger in pitch excursion than rising ones). Those biases were present at the most difficult excursions (near threshold) leading to the conclusion that speech strategies are employed when the non-speech context is highly demanding (Peretz et al., 2011). Response biases for falling and rising pitch contours have been found before in Mandarin speakers and were interpreted as above in the framework of statistical learning (Bent et al., 2006). The evidence might thus seem conflicting, since tone-language experience sometimes enhances pitch perception while at other times it poses limitations or biases. Nonetheless, these findings are consistent with the fact that linguistic experience shapes sound processing either by enhancing or by restricting it depending on the specific sound attribute and the level of processing studied.

It is also of interest to examine the consequences of sound perception deficits. Individuals with tone deafness have difficulties in fine-grained pitch discrimination, particularly detecting pitch changes smaller than one semitone. This deficiency cannot be attributed to lack of musical training, brain lesions (which differentiate Congenital Amusia [CA] from acquired amusia), low IQ or level of education, hearing impairment, or another identifiable neurological or psychiatric disorder (Steward, 2008). Are these pitch deficits specific to music or are they domain-general?

New findings suggest that the deficit is not as domain-specific as it was originally thought to be, since individuals with tone deafness show impaired linguistic pitch perception. Their ability to discriminate pitch variation in an unfamiliar language, namely Mandarin, is significantly worse than that of controls (Nguyen et al., 2009). This finding suggests that lexical tone discrimination is mediated by the same (in this case impaired) pitch system as music (Nguyen et al., 2009). Impaired pitch processing has been found at a suprasegmental level as well. Tone deaf individuals fail to differentiate statements from questions when intonation is the only source of information they can rely upon (Liu et al., 2010). Furthermore, they appear to have phonological and phonemic awareness deficits, deficits that lie outside the narrow domain of music (Jones et al., 2009).

It was not until recently that the incidence of tone deafness in tone language speakers was examined systematically. One of the main findings is that tone deafness does occur in tone language speakers, despite the fact that in principal they should be more “trained” with processing fine-grained pitch information (Jiang et al., 2010; Nan et al., 2010). What is striking is that some tone deaf Mandarin speakers also have difficulties discriminating Mandarin tones (Jiang et al., 2010; Nan et al., 2010). These individuals confuse lexical tones in words and also fail to discriminate between statements and questions, thus exhibiting both segmental and suprasegmental pitch processing deficits (Jiang et al., 2010). Although these deficits arise mostly in laboratory conditions (Liu et al., 2012), lexical tone and intonation difficulties in Mandarin speakers suggest that the disorder has domain-general consequences. Tone deafness is thus a domain-general rather than purely musical disorder, a fact that offers support for theoretical frameworks which propose common auditory processing mechanisms for music and language.

### Subcortical and cortical evidence

At the subcortical level, results show domain-general pitch processing benefits arising from domain-specific experience with language. In one such experiment, tone language speakers' FFR responses to pitch changes were compared to non-tone language speakers, musicians and non-musicians (Bidelman et al., 2011a). Results showed that experience with linguistic pitch enhanced FFR encoding of musical pitch patterns. Despite the fact that there was an influence of domain on the features extracted from pitch patterns in the study, there was nonetheless transfer between domains suggesting that brainstem neurons are amenable to plastic changes and that this has domain-general consequences.

Interestingly, neuroplasticity in pitch processing at this subcortical level of sound encoding is not restricted to the domain in which pitch contours are relevant (Krishnan et al., 2010a,b). Strong effects of context which arise in other studies (see Nan et al., 2009 and Tervaniemi et al., 2009) do not seem to influence brainstem responses. This finding led Krishnan et al. (2010b) to conclude that language and music are “epiphenomenal” with respect to subcortical pitch encoding and that the encoding mechanism has evolved to capture information in the acoustic signal that is of relevance in each domain, in order to facilitate higher-order cortical processing of pitch across domains.

The question that arises, however, is whether enhanced subcortical encoding of pitch has any consequences for musical pitch perception at a behavioral level. In order to provide an answer, Bidelman et al. (2011b) compared Mandarin speakers, musicians and non-musicians' FFR responses and perceptual discrimination performance using musical pitch stimuli. They found that tone language experience enhances subcortical pitch processing in a manner similar to musical experience. However, this was not evident at a behavioral level. Although Mandarin speakers performed better than non-musicians, the FFR response accuracy was a successful predictor of behavioral performance only for the musician group. Thus, while subcortical pitch encoding is sharpened in tone language speakers, this is a necessary but not sufficient condition for perceptual advantages to occur in behavior (Bidelman et al., 2011b).

Evidence concerning cortical processing suggests that language experience can have the same advantageous effects as music in processing pitch in domain-specific or domain-general contexts. Chandrasekaran et al. (2007a) tested Mandarin and English speakers using an oddball paradigm with Mandarin tones, and found that the MisMatch Negativity (MMN) elicited by the Mandarin speakers was significantly larger in amplitude. This result suggests that long-term experience with linguistic pitch patterns will enhance processing of similar pitch patterns at a cortical preattentive level. This holds even when non-speech homologues are used, as long as they preserve the language relative pitch pattern (Chandrasekaran et al., 2007b). What is also of great interest is the fact that experience with linguistically relevant acoustic information such as phoneme duration, which is important in some languages, can generalize to perception of sound duration in a non-linguistic context (Tervaniemi et al., 2006; Marie et al., 2012).

In an investigation of the electrophysiological responses to pure tones presented in a discrimination task and a pitch interval discrimination task, it was shown that tone language experience influenced the timing of the neuronal response to pitch differences (earlier in tone language speakers), and the distribution of processing (more focal in tone-language speakers and more widely distributed in non-tone-language speakers) (Guiliano et al., 2011). Finally, a study, using a refined design, directly compared the effect of tone language and music experience in the preattentive processing of pitch contours resembling those of tone languages (Chandrasekaran et al., 2009). Mandarin native speakers were compared to English speaking musicians and English speaking non-musicians using Iterated Rippled Noise (IRN) stimuli (iterations of adding a delayed copy of white noise sample to itself which produces a pitch sensation) to create dynamic pitch trajectories that were analogues of lexical tones but lacked the formant structure of real speech (Chandrasekaran et al., 2009). The stimuli included between- and within-tone category conditions to control for categorical perception vs. auditory perception effects. Mandarin speakers had significantly larger MMN responses than musicians and non-musicians in both conditions, while musicians had significantly larger MMN responses than non-musicians. No categorical perception effects were evident at the preattentive level in Mandarin speakers. These results demonstrate that there is experience dependent auditory cortical plasticity that generalizes from specific experiences to domain-general abilities, but also that this plasticity remains more sensitive to the specific context in which it was acquired.

The neural correlates of tone deafness can also help to elucidate the cortical processing of speech and music. Tone deaf individuals' electrophysiological responses to inappropriate intonation during speech intonation differ significantly from those of normal individuals (Jiang et al., 2012). Whereas appropriate vs. inappropriate intonation elicits N100 and P600 ERP effects in control participants, such effects are absent in tone deaf participants (Jiang et al., 2012). The absence of a P600 effect in detecting incongruence between linguistic syntax and intonation is reminiscent of the absence of the same effect when incongruence between a note and its tonal context (musical key) fails to be detected in the same group (Peretz et al., 2009). These electrophysiological findings are in accordance with behavioral data (see section Behavioral evidence) and strongly suggest that there is an overlap in neuronal resources used for speech and music.

Although an fMRI study on speech processing and tone deafness has yet to be conducted, evidence from the music domain show abnormal activations to pitch changes in fronto-temporal areas (Hyde et al., 2011). In order to find which node in this fronto-temporal network is underlying the pitch perception-production deficits observed in tone deafness, transcranial direct current stimulation (tDCS) was used to selectively “block” activation in specific brain areas (Loui et al., 2010). Inferior frontal and superior temporal areas were interrupted with tDCS in normal participants during a pitch perception and production task. The results revealed that the left posterior inferior frontal gyrus (IFG) and the right posterior superior temporal gyrus (STG) stimulation affected performance most strongly. When these areas are interrupted, the pitch performance profile of normal individuals resembles that of tone deaf individuals (Loui et al., 2010). Interestingly, these areas seem to be part of a shared network for processing pitch in language and music in Mandarin-speaking musicians. Nan and Friederici (2012) found that in these individuals, who have extensive experience with pitch in both domains, processing pitch incongruities engages the right STG and the left IFG (BA 45). While the right STG is thought to be involved in perceptual pitch processing, the left IFG is responsible for processing pitch at a higher cognitive level irrespective of domain.

To summarize, neural evidence seems to support the view that resources between language and music are shared. Key stages of auditory processing, ranging from subcortical pitch encoding in the inferior colliculus to higher order pitch pattern representation in the STG, are modulated by linguistic experience in a way comparable to music experience. This is in agreement with common processing mechanism accounts. Moreover, the fact that the strongest evidence comes from subcortical sources indicates that bidirectional effects are more prominent in early auditory stages where the auditory signal is processed independent of its linguistic or musical function.

### On-line speech and music processing interactions

As we have seen in the previous sections, speech and music processing are inter-dependent, at least over time (musical experience shapes later linguistic processing, and language experience shapes later musical processing). These inter-dependencies are open to two interpretations, however. One possibility is that speech and music compete for the same resources but remain independent processes. The other possibility is that they rely on the same resources but are actually processed concurrently, in an integrated, holistic way. In order to investigate these two alternatives, one has to look at instances where music and speech are processed simultaneously, as in sung speech.

To investigate simultaneous processing of speech and music, Kolinsky et al. (2009) conducted a speeded classification experiment where participants heard two non-words, differing in their last vowel, sung on an ascending or descending interval. Participants were asked to classify the stimuli according to a specified dimension: melodic (ascending or descending interval), or phonological (according to vowel identity). They were much faster in their classifications when the two dimensions varied in a redundant way (when pitch interval and phoneme identity varied consistently together), and much slower when the variation was orthogonal (when both dimensions varied inconsistently), compared to baseline (when only the task relevant dimension varied). This is evidence that the two dimensions interact; participants could not filter out irrelevant variations in one dimension when processing the other, while, importantly, they gained in performance when this variation was redundant, indicating that the two are processed integrally (Kolinsky et al., 2009). Note, however, that although integrality was observed for vowels and pitch intervals, it was not found when the vowels were replaced by consonants.

Recent MEG and EEG data support the shared pitch-vowel processing evidence, by showing that the source of increased neuronal response to vowels compared to non-vowels coincides with the source of increased activation to pitch compared to non-pitch stimuli (Gutschalk and Uppenkamp, 2011). This common source was identified as the antero-lateral HG in the Superior Temporal Plane. The same region showed a selective adaptation effect to vowel identity, placing at least part of vowel perception as early as in the PAC (Gutschalk and Uppenkamp, 2011).

This language-music interference effect was also found in a task with real words sung on simple melodies. It took participants significantly longer to judge whether two words or two melodies are the same, when the irrelevant dimension would vary within pairs (Gordon et al., 2010). As in the Kolinsky et al. (2009) study, asymmetric interference was found, with more interference from word processing on melodic judgments than the other way around (Gordon et al., 2010).

Following up on these results, Lidji et al. (2009) examined whether the vowel-interval interaction occurs preattentively. If pitch and vowels are processed independently, then a MMN ERP response to a simultaneous deviation in both attributes should have amplitude equal to the sum of the MMN ERPs elicited to each one respectively. What they found was that the MMN amplitude to the simultaneous (double deviant) manipulation of vowel and pitch was not additive, providing evidence for the interaction and not the independence account (Lidji et al., 2009). The same interaction was found for consonant-pitch double deviants' elicited MMNs, suggesting that, at a preattentive level, consonants are also processed by the same resources as pitch (Gao et al., 2012). Furthermore, Gordon et al. (2010) report that the amplitude of the electrophysiological responses to double deviant pairs of sung real words are not additive, as the independence account would have predicted. Moreover, the different melody condition elicited a negativity component (300–500 ms), very similar to the N400 in the different word condition. It was suggested that this might denote violations of “semantic” expectations induced by change in music comparable to semantic violations in language.

The interaction account is also supported by fMRI data. When participants are asked to pay attention to music (simple melodies) and language (real words) simultaneously in sung stimuli, the interaction employs a bilateral network including the middle and superior temporal gyri, the insula, the anterior and posterior cingulates, and the inferior frontal gyri (Schön et al., 2010). Interestingly, there is a quantitative rather than a qualitative difference between the cerebral networks involved in speech and song processing (Schön et al., 2010; Tierney et al., 2012). In an fMRI adaptation study, the left mid-STS showed greater adaptation when lyrics and music were repeated compared to conditions where at least one of them differed (Sammler et al., 2010). Activation to song seems to be following a continuous processing course, with more integrated sound processing occurring in the mid-section, and more domain-specific processing of lyrics in the anterior section of the STS (Sammler et al., 2010).

Song has been described by Peretz (2009) as a “natural alliance” between language and music. It has been also suggested that singing might have played an intermediate role in the evolution of language in humans (Masataka, 2007). We have just reviewed results from studies looking at this music-language alliance in order to shed more light on the underlying processes involved when speech and music sounds are processed simultaneously. The evidence is in favor of interaction, at least up until the level of phonetic perception of speech. Indeed, experiments focusing on the interaction at the level of melodic and semantic processing failed to find evidence for interactions (Besson et al., 1998; Bonnel et al., 2001). Processing of sung speech results in behavioral and neural effects that are not equal to the sum of the effects of lyrics and melody separately.

Although this section is devoted to interactions observed during on-line processing of music and language, it is worth mentioning that there is also evidence in favor of interaction from offline, long-term experience effects. We have already mentioned results showing an interaction between music training and native language representations in non-native speech perception (Sadakata and Sekiyama, 2011). Another study has examined the interactive effects of musical and linguistic experience by looking at how these different experiences affect learning an unfamiliar tone language. Cooper and Wang (2012) tested tone identification and sound-to-meaning learning performance in English-speaking musicians and non-musicians as well as in Thai-speaking musicians and non-musicians. If the effects of musical and linguistic experience were independent and linearly additive, Thai musicians should perform best given that they have both types of experience. On the contrary, the Thai-speaking musicians not only were outperformed by the English-speaking musicians in both tasks but were also outperformed by the Thai non-musicians in the sound-to-meaning learning task. These findings demonstrate that, in isolation, musical and linguistic experience has beneficial effects on tone identification and sound-to-meaning mapping. However, in individuals who have acquired both types of experience, such as Thai musicians, music and language interact: the beneficial effect of music is restrained by interference from the native language on the non-native tones and the beneficial effect of language is in turn restrained by music interference. While English speakers simply relied on low level sound processing, which was enhanced in those who were musicians, Thai speakers could not prevent interference from higher level processing calling on tone categories from their native language. The study confirms that there is dynamic interplay of linguistic and non-linguistic pitch experience in tone perception.

### Overlapping functional and structural correlates of speech and music

Another way of gaining insight to shared resources between speech and music is by investigating shared brain areas and how they are shaped by experience in these two domains. If they employ common neural mechanisms, then we should expect an overlap in the structural consequences of this extensive experience. In this section, we will review findings on two cortical areas important for sound processing in both language and music, the IFG and the Auditory Cortex. The reader should bear in mind that the studies cited have not been conducted so as to directly compare language and music and also that they did not use designs that can fully dissociate functional from structural changes in neuronal populations within a brain region. As Price and Friston (2005) have noted: “there is a many-to-many mapping between cognitive functions and anatomical regions.” While we acknowledge that there are many issues with respect to spatial precision and function-to-anatomy mapping in neuroimaging studies, we still would argue that it is worth examining the function-structure relationship resulting from linguistic and musical experience.

#### The left IFG shaped by language and music

Accumulating neuroimaging evidence suggest that the left IFG serves as a hub for processing structured sequences across language, music, and action (Fadiga et al., 2009). This area is well known to be involved in language, with BA44 and BA6 activated during phonological processing, BA44 and BA45 during syntactic processing, and BA45 and BA47 during semantic processing (Hagoort, 2005). As far as action is concerned, BA44 is part of the mirror neuron network for observation and motor imitation of action (Molnar-Szakacs et al., 2005). As mentioned in section subcortical and cortical evidence, the left IFG is found to be part of a shared language-music pitch network in Mandarin speaking musicians, one that is engaged in cognitive pitch representation processing in both domains (Nan and Friederici, 2012).

Sluming et al. (2002) found that experienced symphony orchestra musicians had increased Gray Matter (GM) density in Broca's area. In a subsequent study, a significant difference between the musicians and controls was observed in the GM of the left Pars Opercularis (POP, BA44) (Abdul-Kareem et al., 2011). Significant positive correlations were found between GM in the left POP and years of music training and performance in the musician group (Gaser and Schlaug, 2003; Abdul-Kareem et al., 2011). These findings can be attributed to extensive action-related sound processing in musicians, involving components of the mirror neuron system (Abdul-Kareem et al., 2011). Conversely, individuals with impaired pitch processing have significantly less gray GM concentration in the left Pars Orbitalis in the IFG (area BA 47) (Mandell et al., 2007) as well as increased cortical thickness in the right homologue of the same area (Hyde et al., 2007). These morphological measures correlated with individuals' performance in musical tasks (Hyde et al., 2007; Mandell et al., 2007).

Golestani et al. (2011) studied the brains of another group of individuals who have extensive experience with sound processing: phoneticians. They found, among other things, that GM volume in the left POP was larger in phoneticians and that the number of years of experience in phonetic transcription could predict successfully the left POP's surface area with a similar trend for the volume measure (Golestani et al., 2011). On the other hand, poor phonetic perceivers of a non-native vowel contrast have more white matter (WM) density in their right POP (Sebastián-Gallés et al., 2012), which could be part of a compensatory mechanism (Wong et al., 2007a).

In sum, the left IFG has greater volume in individuals whose profession requires detailed monitoring, production, and manipulation of music or language sounds, while in individuals with poor sound skills a decrease or an increase in its right homologue is observed. Importantly, volume and surface measures in the IFG correlate with the amount of experience with sound processing as well as the degree to which this is poor or impaired.

#### The role of the auditory cortex in language and music

Naturally, when discussing sound processing in either language or music, the main area of interest is the auditory cortex including the PAC and belt areas in the supratemporal plane. The PAC lies roughly at Heschl's gyrus (HG) and its adjacent sulci although there is big inter- and intra-individual variability (Da Costa et al., 2011). The auditory cortex, specifically the left lateral HG and PT, is engaged in the acoustic analysis of linguistic sounds (Obleser et al., 2007) as well in the production of melodies and sentences (Brown et al., 2006) while the same regions bilaterally are important for pitch processing (Barker et al., 2012). One would therefore expect that experience with linguistic or music sounds would have an effect on the morphology of these auditory regions.

Consistent with this assumption, several studies report greater GM density in Heschl's Gyri of musicians (Schneider et al., 2002; Gaser and Schlaug, 2003; Bermudez et al., 2009). Schneider et al. (2002) found that GM volume in the anteromedial HG bilaterally was larger in both professional and amateur musicians compared to non-musicians, with the total volume of the right HG being larger in professional musicians only. The anatomical differences in the amHG were positively correlated with participants' neurophysiological responses to pure tones as well as musical aptitude measures (Schneider et al., 2002).

By performing a whole-brain volumetric analysis in male keyboard players, Gaser and Schlaug (2003) found that GM volume in the left HG differed according to musician status (naïve, amateur, professional), while both gyri showed significant differences in a more liberal threshold in agreement with Schneider et al. (2002). In a less homogeneous group of musicians, Bermudez et al. (2009) found differences in GM in the right posterolateral HG. GM density in the right PAC also correlates with relative pitch judgment performance in a music transformation task in individuals with variable musical training (Foster and Zatorre, 2010). Increased volume in the right HG after receiving instrumental training has further been reported in children using a longitudinal design with random assignment of children to training conditions (Hyde et al., 2009). This increase correlated with behavioral measures of melodic and rhythmic abilities (Hyde et al., 2009).

Bermudez et al. (2009) also performed a cortical thickness analysis that revealed greater cortical thickness in the PT (BA 42, posterior to PAC) bilaterally in musicians. A previous study measuring GM volume had found that the right PT and Planum Polare (PP) (BA 52, anterior to PAC) had significantly greater GM density in musicians (Bermudez and Zatorre, 2005). Interestingly, tone deaf individuals have less GM in the left STS (adjacent to PT) although there is no correlation between this morphological measure and pitch performance (Mandell et al., 2007). However, cortical thickness in the right STG (close to BA 22) does correlate negatively with music pitch performance with tone deaf individuals having significantly greater thickness in that region (Hyde et al., 2007).

In the search for neuroanatomical markers of experience with a tone language, Crinion et al. (2009) compared Chinese speakers (both native and L2 learners of Chinese to control for ethnicity) to multilingual non-Chinese speakers. Regions in the auditory cortex, specifically the right PP in the anterior superior temporal lobe showed significantly more GM in Chinese speakers (Crinion et al., 2009). Greater WM density was found in the right HG and just posterior to the left HG in phoneticians (Golestani et al., 2011). Heschl's gyri were reportedly larger in phoneticians, while gyrification was greater in the left but not the right hemisphere compared to controls. Neither volume nor gyrification correlated with phonetic transcription experience, leading to the conclusion that the morphology of this structure is innately defined (Golestani et al., 2011). However, a recent study contradicts this conclusion. By looking at early Spanish-Catalan bilinguals who learn to master two different phonological systems from birth, Ressel et al. (2012) found that bilinguals had greater GM and WM density in both Heschl's gyri. Since, contrary to phoneticians, bilinguals cannot be self-selected, it is assumed that there is a causal link between language experience and HG differences (Ressel et al., 2012).

HG structure also correlates with learning new linguistic sounds. Performance in a “Mandarin-like” word learning task correlated positively with gray and WM density in the left HG (Wong et al., 2008). Successful learners had larger left HG volume and learning speed correlated with GM in the left HG as well (i.e., the faster the learning, the greater GM) (Wong et al., 2008). Apart from linguistic pitch, when learning a non-native phonetic contrast, fast learners have increased volume and WM density in the left HG (Golestani et al., 2007).

To conclude, despite the differences between the samples recruited, the measures used and the analysis methods between these studies, their results suggest that morphological differences in auditory areas constitute structural correlates of language and music aptitude and experience or lack thereof.

#### Summary

Music and language expertise appear to correlate with differences in brain anatomy, especially in regions that play an important role in sound processing. As with most neuroanatomical studies, there are two caveats in interpreting the results. The first one is related to causal links between brain structure and experience. Given the fact that there is great inter-individual variability in the regions discussed, and that it is very difficult to control for those prior to training initiation in expert individuals, self-selection cannot be ruled out. That is, individuals with greater HG surface might have a propensity to be better sound learners and become musicians or phoneticians. Although there are evidence against self-selection (see Hyde et al., 2009 and Ressel et al., 2012) it remains an open question whether the structural differences observed in IFG and PAC are the cause or the effect of musical and/or linguistic experience. An experimental way to surpass this obstacle is by conducting longitudinal studies where participants are randomly assigned to music training.

The second caveat lies in the sort of arguments presented by Price and Friston (2005). Gray or WM density, volume, and cortical thickness constitute quite crude measures of brain plasticity. They cannot dissociate quantitative (same neuronal populations but different degree/number that light up) from qualitative (dissociable neuronal populations) differences as the mechanisms underlying plasticity changes. We therefore ought to be cautious when claiming that the same regions are being shaped by music and speech. Even if the exact same anatomical regions show changes with both types of training without knowing the underlying mechanism we might be looking at independent phenomena (different neuronal populations that are shaped by music and speech but lie within the same anatomical region). Neuroanatomical evidence needs to be combined with more sensitive measures looking at functional activation differences, for example using multivariate pattern recognition methods in fMRI data (see Staeren et al., 2009).

## Challenges in looking at the equation from the language perspective

Having presented evidence in favor of bidirectional influences between language and music, let us consider the main challenges or limitations when looking at the language-music equation from the perspective of effects of linguistic experience.

First, what constitutes “language experience”? This is one of the major methodological challenges in this research area. What kind of experience with linguistic sounds can qualify as being comparable to music training? A plethora of studies have focused on tone language speakers, mostly due to the fact that tone languages primarily make use of pitch in order to convey lexical information. Since pitch is a sound property that is shared between language and music, tone language speakers have been regarded as comparable to musicians. Speakers of quantity languages, in which vowel duration information plays an important role, have been studied as well with respect to their sensitivity to sound duration in non-linguistic contexts (Tervaniemi et al., 2006; Marie et al., 2012). Early bilinguals have also been considered to have special linguistic experience based on the fact that they have learned to manipulate different phonetic inventories from an early age on (Krizman et al., 2012; Ressel et al., 2012). Other candidate populations include professional phoneticians, simultaneous interpreters, and multilingual individuals, with different advantages and disadvantages for each group.

This methodological difficulty is in fact two-fold, as the lack of a strict definition for language experience leads to great heterogeneity in the populations recruited. Contrary to musicians, where heterogeneity, though of course also present in the wider population, can at least be controlled within an experiment (for example one can recruit pianists from a specific conservatory, following the same curriculum and training, having achieved the same level of performance etc.), all the aforementioned linguistic groups differ fundamentally in their expertise, making experimental control very difficult. Acquisition of expertise is in some cases achieved implicitly, by exposure to speech input (in the case of tone or durational language speakers, and in bilinguals), while in other cases it is achieved explicitly, by formal training (in the case of phoneticians and simultaneous interpreters). As a result, the level of linguistic expertise cannot be defined as systematically as in musicians. Lastly, in each group a set of distinct sound properties are “trained” more than others and this increases the difficulty of making appropriate comparisons or predictions.

Experimental designs are affected substantially by this heterogeneity. This means that finding tasks and measures that are “fair” or sensitive enough to capture any advantages of language experience on sound processing is not an easy endeavor. For example, there are studies reporting enhanced sound processing in tone language speakers in electrophysiological measures in the absence of behavioral advantages (Bidelman et al., 2011b; Guiliano et al., 2011). Finding measures that are sufficiently sensitive depends not only on the heterogeneity of the groups under investigation but also on the fact that the effects that are being investigated are likely to be quite small.

The selection of stimuli is also crucial, especially when comparing “language experts” with musicians. Let us take, for instance, studies that focus on tone language speakers' pitch perception abilities. It has been consistently shown that the context in which pitch stimuli are embedded influences their processing (Nan et al., 2009; Bidelman et al., 2011a). Pitch information can serve multiple functions in language (lexical, syntactic, prosodic and/or pragmatic information) compared to music, and the context can bias its perception and neural processing accordingly. Finding “context-free” pitch stimuli is difficult but imperative in order to achieve an objective assessment of the effect of language experience on pitch processing. Such attempts have been made with respect to pitch (see e.g., the IRN in Chandrasekaran et al., 2007a) but not to other sound properties. Of course, language is more than tones, as music is more than pitch intervals. Both domains are multi-faceted and thus hard to parse or fit into neat categories without sacrificing their richness and ecological validity.

Another major difficulty when looking at linguistic experience and how it might affect sound perception is the extent to which this experience taps into or “trains” top-down processing mechanisms. According to the Reverse Hierarchy Theory (Ahissar et al., 2009) perception is by default guided by higher-order mechanisms, leading to divergence or convergence of low-level information into higher-order categories. Perceptual attunement depends on the engagement of higher-order cortical structures that search backwards for the most informative low-level population with respect to the task in hand (Ahissar et al., 2009). Perceptual learning is therefore taking place when the signal to noise ratio from lower level input increases as a function of attention and training. Music experience triggers top-down mechanisms, since attention and purposeful repetition are essential elements of music training (Patel, 2011). In contrast, when acquiring one's native language, little explicit focus is placed on phonology and other sound properties of the speech signal (contrary to what's happening when learning a second language). This explicit training to pay attention to sounds offers a great advantage to musicians over tone language speakers, for instance.

Perceptual attunement is not the only benefit music training offers. Other higher-order cognitive functions such as auditory working memory, IQ, and executive functions are also enhanced in musicians and contribute to their behavioral performance superiority (Schellenberg, 2004, 2006; Strait et al., 2010; Degé et al., 2011; Moreno et al., 2011). Although it is an empirical question whether this is also true for tone language speakers, there are few theoretical reasons to assume that this is the case (though see Bidelman et al., 2013).

A solution to the problems associated with explicit training would be to focus on individuals with linguistic experience that has been acquired involving top-down mechanisms. Early bilinguals or multilinguals could be an example of such individuals. It is top-down processing in bilinguals (Rodriguez-Fornells et al., 2006) that makes a difference in their sound processing abilities compared to monolingual tone language speakers. Recent findings have shown that bilinguals are less susceptible to the distorting effects of background noise when listening to speech (Krizman et al., 2012), something that has been consistently shown in musicians (Parbery-Clark et al., 2009) and children receiving music training (Strait et al., 2012). Similarly to musicians (Strait et al., 2010), these beneficial effects of bilingualism could be mediated by enhanced top-down mechanisms such as auditory cognitive abilities and executive functions (Krizman et al., 2012) When one has to reflect on language sounds and to learn to dissociate, manipulate, and inhibit different sound systems from a sensitive period on, more top-down processing involvement would be expected. The same would also hold for phoneticians or interpreters, who have extensive linguistic experience more comparable to purposeful music training.

Despite all the aforementioned challenges, we believe that this line of research should continue. One cannot have a complete account of the effect of music on language unless the inverse effect is also systematically studied to inform existing theoretical frameworks.

## Conclusion: speech and music in interaction

We have reviewed the literature on music and speech, by taking a less common stance and focusing primarily on the effect of language experience on music, or, more correctly, on sound processing. We have presented behavioral, electrophysiological, and neuroimaging data revealing the effects of language experience on music and sound processing, and evidence of on-line interactions across domains, and we have presented findings on associations between experience in the two domains and differences in brain structure. Consistent with a shared auditory skills account, language experience shapes sound perception, by augmenting it or in some cases restricting it. Building up on the shared auditory skills framework, we reviewed the literature on tone deafness and saw that this impairment affects both musical and linguistic pitch processing. Data on song processing added to the picture of what is actually shared when linguistic and music sounds are processed simultaneously, while neuroanatomical data was presented on the infrastructure involved in both domains. Furthermore, we have seen that experience with pitch in a linguistic context can enhance music pitch processing. In other words, there can be positive transfer from the speech domain to music, as defined by Besson et al. (2011a,b). Additionally, evidence for enhanced subcortical pitch encoding in tone language speakers suggests that language experience can, under certain circumstances, meet the OPERA hypothesis requirements (Patel, 2011).

Evidence of language on music effects is sparser than of the reverse. There is need for more research to broaden our understanding of bidirectional language-music effects. For example, the “Shared sound category learning mechanism hypothesis” (Patel, 2008) has not yet been addressed from the language perspective, to the best of our knowledge. Future research aiming to test this hypothesis will need to look into whether learning music categories might be modulated by linguistic experience or expertise. The existing frameworks should also try to accommodate observed phenomena. For instance, in some cases, we have seen that although neuronal sound mechanisms show a clear language experience advantage in performance, no such advantage exists in behavior (Bidelman et al., 2011b). The same pattern has been observed in musician studies (Ott et al., 2011). The theoretical accounts do not yet make predictions about these differences.

There are many other missing pieces in this puzzle. What we wanted to demonstrate, however, is that some of the pieces can only be revealed by looking at the effect of language experience on sound processing. We hope that this review will motivate future research that considers the effects of both linguistic and musical experience, as well as their mutual interactions.

The existing data, however, already offer strong support for a shared auditory skills account of speech, music, and sound processing (Patel, 2008, 2011; Kraus and Chandrasekaran, 2010; Besson et al., 2011a,b). In particular, the evidence points to a synergistic account: music and linguistic experience influence sound processing beyond their narrow domains, and while doing so they mutually interact. As Zatorre and Gandour (2008) have suggested, the synergy probably lies in the interplay between the sensory encoding of sound and the abstract representation of sound, that is, between domain-general, low-level acoustic processes and domain-specific, higher-level cognitive processes. Synergy at this stage of processing would result in the four bidirectional phenomena that have been reviewed: Interactions over time, where prior music experience influences current linguistic behavior and prior language experience influences current musical behavior; interactions across domains in on-line processing; shared underlying brain structures; and sub-cortical and cortical changes shaped by speech and music experience, acting in concert.

### Conflict of interest statement

The authors declare that the research was conducted in the absence of any commercial or financial relationships that could be construed as a potential conflict of interest.
